# Medical image fusion with deep neural networks

**DOI:** 10.1038/s41598-024-58665-9

**Published:** 2024-04-04

**Authors:** Nannan Liang

**Affiliations:** School of Informatics and Engineering, Suzhou University, Suzhou, 234000 China

**Keywords:** Medical research, Engineering, Mathematics and computing

## Abstract

Medical image fusion aims to fuse multiple images from a single or multiple imaging modes to enhance their corresponding clinical applications in diagnosing and evaluating medical problems, a trend that has attracted increasing attention. However, most recent medical image fusion methods require prior knowledge, making it difficult to select image features. In this paper, we propose a novel deep medical image fusion method based on a deep convolutional neural network (DCNN) for directly learning image features from original images. Specifically, source images are first decomposed by low rank representation to obtain the principal and salient components, respectively. Following that, the deep features are extracted from the decomposed principal components via DCNN and fused by a weighted-average rule. Then, considering the complementary between the salient components obtained by the low rank representation, a simple yet effective sum rule is designed to fuse the salient components. Finally, the fused result is obtained by reconstructing the principal and salient components. The experimental results demonstrate that the proposed method outperforms several state-of-the-art medical image fusion approaches in terms of both objective indices and visual quality.

## Introduction

Image fusion is an effective technique which can fully integrate the complementary information from multiple sensors or different aspects. This technique has been widely applied in many fields, such as land cover mapping^1,2^, object detection^3–5^ and medical diagnosis^6–8^. Medical image fusion is to synthesize multiple medical images from single or different imaging devices. The main purpose of the medical image fusion is to improve imaging quality while preserving the specific features for accurate diagnosis and treatment, which plays an important role in surgical navigation, routine staging, and radio-therapy planning of malignant disease^9–14^.

Currently, medical image fusion mainly focuses on computerized tomography (CT), magnetic resonance imaging (MRI), single-photo emission computed tomography (SPECT) modalities, and positron emission tomography (PET). CT image can clearly reflect main structures, e.g., bones and implants. MRI image records high-resolution anatomical details for soft tissues. However, the MRI image is not sensitive to the diagnosis of fractures compared to CT image. Moreover, PET image has the property of high sensitivity due to the molecular imaging technique, but it is with lower resolution. SPECT image is utilized to study the blood flow of tissues and organs by nuclear imaging technique. Therefore, each modality has different merits and drawbacks. In order to fully integrate the advantages of different modalities, an effective scheme is to fuse the complementary information from multi-sensor images^15–20^.

To date, various medical image fusion approaches have been proposed, which can be divided into two types: spatial domain fusion and transform domain fusion. The spatial domain methods usually adopt a pixel or block-based fusion strategy^21^, in which the source images are fused with a designed activity level measurement such as spatial frequency and sum-modified-Laplacian. For instance, Li et al. proposed a matting-based image fusion approach to achieve image fusion^22^, in which the image matting technique is used to decide the multi-focus regions. In Li’s paper^22^, a wise-block medical image fusion method was proposed. Source images was divided into smaller blocks according to local window. The average value of principal components of all the blocks was used as the weights.

In contrast to the spatial domain approaches, the transform domain approaches mainly contain three key steps. First, the original images are decomposed into the transform domain via a wavelet transform. Then, the designed fusion rule is used to fuse the transformed coefficients. Finally, an inverse transform is performed on the fused coefficients to obtain the fusion result. Multi-scale transform (MST) approaches have become the very popular research topic, such as curvelet transform (CVT)^23^, non-subsampled contourlet transform (NSCT)^24^, non-subsampled shearlet transform (NSST)^25^, and shift-invariant dual-tree complex shearlet transform (SIDCST)^26^. For example, Bhatnagar et al. developed a general fusion framework for multimodal medical images based on NSCT^24^. Yin et al. proposed a decision fusion rule to achieve medical image fusion in NSST domain^27^, in which four different types of medical image fusion problems were used to verify the effectiveness. In Yin’s paper^26^, a SIDCST was constructed by cascading the dual-tree complex wavelet transform (DTCWT) and shearlet filters, which makes full use of the limited redundancy of the DTCWT and the directional selectivity of the shearlet filter.Besides, sparse representation (SR) methods have been widely researched in image fusion field^28^. For example, Yang et al. first applied the SR in image fusion field, which obtains satisfactory performance^28^. Liu et al. proposed a general fusion framework by combining the SR and MST so as to improve the details of fusion result^29^. Liu et al. introduced a convolutional sparse representation (CSR) into image fusion to overcome the limited ability in detail preservation caused by SR method^30^. These publications have been demonstrated the SR is able to achieve the image fusion.

In recent years, deep learning has become a hot topic in image processing field. Several deep models have been applied in image fusion^31–34^. For instance, in Liu’s paper^31^, the multi-focus image fusion problem is regarded as binary image classification, and the convolutional neural network (CNN) is used to learn the weight map from a large number of labeled training samples. Li et al. proposed a novel deep learning architecture for infrared and visible images fusion problem via combining convolutional layers, fusion layer and dense block^32^. These methods demonstrated that the deep learning model is an effective tool to achieve image fusion.

However, although traditional methods can achieve medical image fusion, the issue of how to utilize CNN effectively for medical image fusion is still an open question. In this paper, a novel medical image fusion method is proposed based on a pre-trained CNN model, called deep medical image fusion (DMIF). First, the source images are decomposed into the principal and salient components via low rank representation. Then, the pre-trained CNN model is utilized to extract the deep features of principal components, and a weighted-average strategy is adopted to fuse the deep features. Second, the salient components are fused with a sum strategy. Finally, the principal and salient components are reconstructed to obtain the fusion result. Experimental results demonstrate that the proposed method can obtain outstanding performance against several state-of-the-art approaches.

The main contributions of this work are shown as follows:

1. A novel medical image fusion approach based on CNN is proposed. The proposed method opens new opportunities for the medical image fusion.
2. Low rank representation is adopted to decompose the medical images into two components for the first time, i.e., principal and salient layers, which is able to effectively separate the structure information and detail information.
3. Experiments on 44 pairs of medical images verify that the fusion performance obtained by the proposed method outperforms other approaches.

The remaining of this work is organized as follows. In Section “Proposed method”, we introduce the proposed method in detail. Section “Experiments” presents the experiment results and analyses. Finally, Section “Conclusions” gives the conclusions of this work.

## Related work

VGG-16^35^ is a convolutional neural network (CNN) architecture designed for image classification. It was proposed by the Visual Geometry Group at the University of Oxford and was one of the top-performing models in the ImageNet Large Scale Visual Recognition Challenge (ILSVRC) in 2014. The architecture is characterized by its depth, utilizing 16 weight layers, named as VGG-16. The network consists of 16 weight layers, including 13 convolutional layers using 3*3 kernel with a stride size of 2 and 3 fully connected layers. The convolutional layers are followed by max-pooling layers using 2*2 filters with a stride size of 2 to reduce spatial dimensions. After each pooling layer, the size of the feature map is reduced by half. The last feature map before the fully connected layers is 7*7 with 512 channels. Despite its simplicity and straightforward architecture, VGG-16 has demonstrated strong performance on various computer vision tasks and served as a foundation for more advanced architectures. Due to the strong feature representation ability, the VGG-16 is considered as feature extractor in this work.

## Proposed method

**Figure 1:**
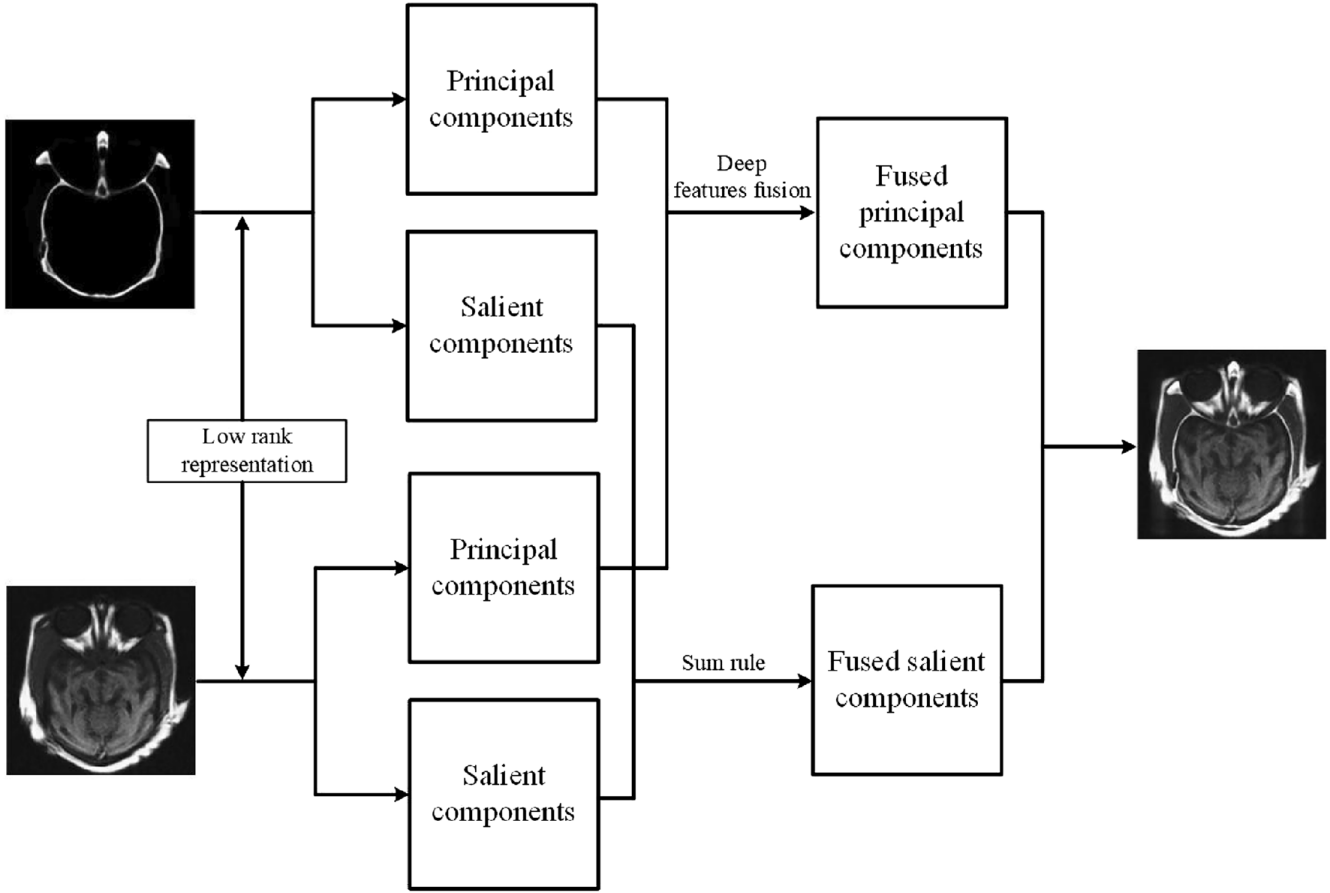
Schematic of the proposed medical fusion method. The principal components record the global structure information and brightness information of the original images. The salient components the local detail information.

Figure 1 shows the flow chart of the proposed fusion framework, which consists of three steps: First, the original images are decomposed into principal and salient components. Second, two different fusion rules are proposed to achieve two component fusion, respectively. Finally, the fused principal and salient components are reconstructed together. The details of our method are shown as follows:

### Low rank representation-based decomposition

Low rank representation (LRR) is to seek the lowest-rank representation among all the candidates that represent all vectors as the linear combination of the bases in a dictionary. Unlike the well-known sparse representation, which computes the sparest representation of each data vector individually, LRR aims at finding the lowest-rank representation of a collection of vectors jointly. Liu et al.^36^ proposed low rank representation to segment data for the first time. However, the LRR fails to preserve the local structure information. Thus, a latent low-rank representation (LatLRR) is proposed, which can extract both the global structure and local details. The LatLRR is formulated as follows:

1 $$\mathop {\min }\limits_{Z,L,E} \quad \left\| Z \right\|_{*} + \left\| L \right\|_{*} + \lambda \left\| E \right\|_{1} ,\,\;s.t.\;X = XZ + LX + E,$$

where $\lambda$ is a free parameter. $\left\| {\, \cdot \,} \right\|_{*}$ is the nuclear norm which is the sum of the singular values of the matrix, and $\left\| {\, \cdot \,} \right\|_{1}$ denotes the $l_{1}$-norm. $X$ is the original image. $Z$ is the low-rank coefficients, and $L$ is the saliency coefficients. $E$ indicates the sparse noisy coefficient. $XZ$ denotes the low-rank part, and $LX$ indicates the saliency part.

Since medical images have high frequency, the low rank representation is adopted in this work. The two source images, denoted as $I_{1}$ and $I_{2}$, are decomposed by the LatLRR to obtain the low-rank part $I_{i}^{r}$ and saliency part $I_{i}^{s}$,$i \in \{ 1,2\}$. The main aim of this step is to obtain the main structure information and spatial details, which is beneficial for preserving the spatial information of source images.

### Fusion of low-rank parts

Low-rank part mainly reflects global structure information and brightness information of the original images. In order to effectively fuse the low-rank parts of original images, the CNN model is adopted to extract the deep features. Specifically, first, the deep features of the low-rank parts are constructed via the pre-trained CNN, i.e., VGG-16. The CNN model can be thought as a composition of a number of functions.

2 $$f(I) = f_{L} \left( {...f_{2} \left( {f_{1} (I;\omega_{1} );\omega_{2} } \right)...,\omega_{L} } \right),$$

where each function $f_{l}$ takes the data samples $X_{l}$ and a filter $\omega_{l}$ as inputs and outputs $I_{l + 1} ,l \in \{ 1,2,...,L\}$, and $L$ is the number of layers.

For the pre-trained CNN model, the filter banks $\omega_{l}$ has been learned from some big dataset, e.g. ImageNet. Suppose the input image $I_{i}^{r}$, the multi-layer features are extracted, which is shown as follows:

3 $$F_{k} = f_{k} \left( {I_{i}^{r} ;\omega_{1} } \right),k \in \left\{ {1,2,3} \right\}.$$

In our work, three convolutional layers, i.e., ‘conv1’, ’conv2’, ’conv3’, are adopted. Then, a block-based average strategy is used to evaluate the weight of each feature.

4 $$\hat{\omega }_{k} (x,y) = \frac{{\sum\limits_{a = - 1}^{1} {\sum\limits_{b = - 1}^{1} {\left\| {F_{k} \left( {x + a,y + b} \right)} \right\|_{1} } } }}{9}.$$

Next, the initial weights are calculated by soft-max operator.

5 $$\hat{\omega }_{k} (x,y) = \frac{{\hat{\omega }_{k} (x,y)}}{{\sum\limits_{k = 1}^{N} {\hat{\omega }_{k} (x,y)} }}.$$

As we all know, the pooling operator in VGG-16 model is a kind of subsampling method, and thus, this operator decreases the spatial size of the feature maps to ${1 \mathord{\left/ {\vphantom {1 s}} \right. \kern-0pt} s}$ times of input where $s = 2$ is the stride of the pooling layer. Thus, in different layers, the size of feature maps is ${1 \mathord{\left/ {\vphantom {1 {2^{i - 1} }}} \right. \kern-0pt} {2^{i - 1} }}$ times of the input image. After we obtain the initial weight map, an upsampling operator is utilized to increase the spatial size of the obtained weights to the one of the source images.

6 $$\begin{gathered} \omega_{k} (X,Y) = \tilde{\omega }_{k} (x,y) \hfill \\ X = \left[ {2^{k - 1} (x - 2) + 1:2^{k - 1} (x - 1)} \right] \hfill \\ Y = \left[ {2^{k - 1} (y - 2) + 1:2^{k - 1} (y - 1)} \right]. \hfill \\ \end{gathered}$$

Finally, the multi-layer features are merged together to obtain the fused low-rank part.

7 $$G_{k} (x,y) = \sum\limits_{k = 1}^{L} {\omega_{k} (x,y) \times F_{k} (x,y)}$$

8 $$G(x,y) = \max \left\{ {G_{k} (x,y)} \right\},k \in \left\{ {1,2,3} \right\}.$$

### Fusion of salient components

The saliency parts preserve the local detail information. Since the saliency features in source images have strong complementary information, a sum rule is adopted to fuse the saliency parts so as to preserve more details.

9 $$H^{s} = I_{1}^{s} + I_{2}^{s}$$

### Image reconstruction

When the fused low-rank part $G$ and saliency part $H$ are obtained, the fusion result will be reconstructed as follows:

10 $$R = G + H,$$

where $R$ is the resulting image.

## Experiments

In order to verify the superiority of the proposed method, several state-of-the-art medical image fusion methods including convolutional neural networks and non-subsampled contourlet transform (CNN-NSCT)^37^, nonsubsampled shearlet transform fusion method (NSST)^38^, nonsubsampled shearlet transform based structure tensor method (NSST-ST)^39^, sparse representation (SR)^28^, multi-scale transform based domain sparse representation fusion method (MST-SR)^29^, guided filtering based fusion method (GFF)^22^, and discrete stationary wavelet transform and an enhanced radial basis function neural network (DSWT-RBFN)^40^, are adopted for comparison. For the MST-SR method, the Laplacian pyramid is used as the multi-scale transform. In addition, the default parameter settings of all compared methods are consistent with the corresponding publications given by the authors.

### Test images

**Figure 2:**
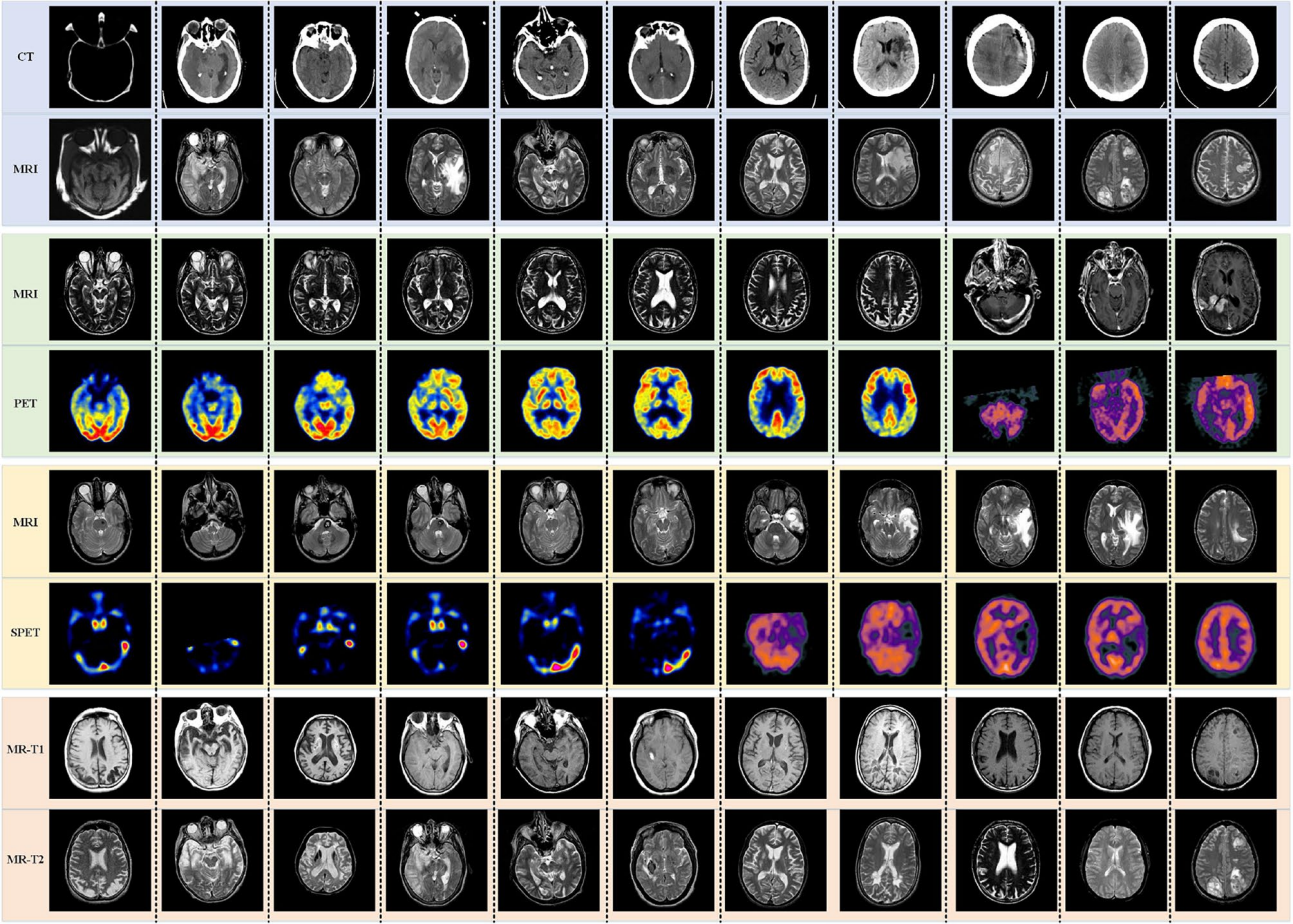
44 pairs of multi-modal medical images.

In our experiment, 44 pairs of multi-modal medical images, i.e. CT and MRI, MRI and PET, MRI and SPECT, MR-T1 and MR-T2, are adopted as experimental datasets. Figure 2 shows the original images, which have been accurately registered before fusion. The size of each source image is 256 * 256 pixels.

### Objective indexes

In order to quantitatively assess the fusion performance of different approaches, several widely used objective quality indexes are adopted in our experiment, including standard deviation (SD)^41^, entropy (EN)^41^, normalized mutual information Q_MI_^42^, and phase congruency Q_PC_^41^. SD calculates the overall contrast of the resulting image. EN measures the amount of information in the fused result. Q_MI_ denotes how much information from source images into the resulting image. Q_PC_ reflects image details in the fused result.

### Experimental results

#### CT and MRI image fusion

**Figure 3:**
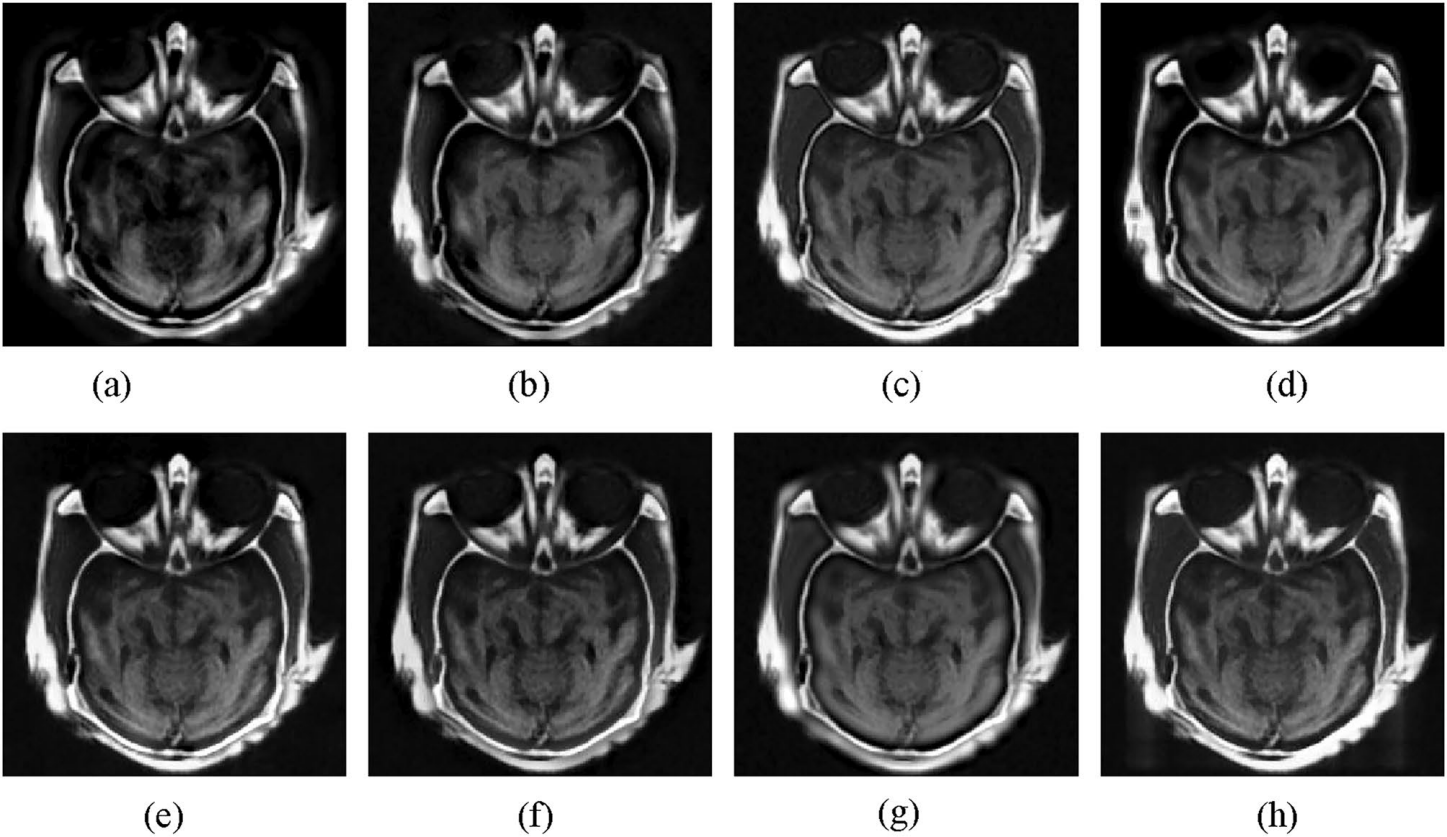
Fusion results of different methods for CT and MRI images. (**a**) CNN-NSCT. (**b**) NSST. (**c**) NSST-ST. (**d**) SR. (**e**) MST-SR. (**f**) GFF. (**g**) DSWT-RBFN. (**h**) Our method.

The first experiment is to fuse CT and MRI images. Figure 3 presents the fusion images obtained by different methods. As shown in Fig. 3, the CNN-NSCT method leads to some loss of local details. The NSST method yields low contrast fusion result. The NSST-ST method fails to well fuse the information of source CT image. The fusion result obtained by SR method appears distortion phenomenon. The MST-SR method suffers from the detail loss of source MRI image. The GFF method decreases the brightness of source MRI image. Although the DSWT-RBFN method retains well the information of original images, the boundaries in the fused image are blurry. In comparison, the proposed method can retain the detail information of original images. In addition, the boundaries in the fused result are visible clearly.

To illustrate objectively the fusion quality of resulting images achieved by different approaches, Table 1 shows the average objective metrics on 11 pairs of CT and MRI images. It can be seen from Table 1 that the proposed approach yields the highest objective indexes in terms of SD, EN, Q_MI_, and Q_PC_, which further proves that the proposed method can effectively achieve the CT and MRI image fusion over other methods. For the computational time, it is show that the running time of the proposed method is acceptable among all studied techniques. Moreover, compared to the CNN-NSCT, the proposed method performs faster.

**Table 1:** Average objective indexes of different methods on 11 pairs of CT and MRI images.

Methods	SD	EN	Q_MI_	Q_PC_	Time(s)
CNN-NSCT	65.87	6.78	0.59	0.56	20.91
NSST	62.77	6.81	0.45	0.51	8.23
NSST-ST	66.28	6.97	0.71	0.55	9.41
SR	67.48	6.45	0.57	0.52	15.34
MST-SR	69.51	6.85	0.53	0.56	17.82
GFF	69.05	6.94	0.69	0.57	**1.35**
DSWT-RBFN	70.12	6.93	0.71	0.54	2.32
Our method	**73.56**	**7.12**	**0.76**	**0.62**	5.61

Significant values are in bold.

#### MRI and PET image fusion

**Figure 4:**
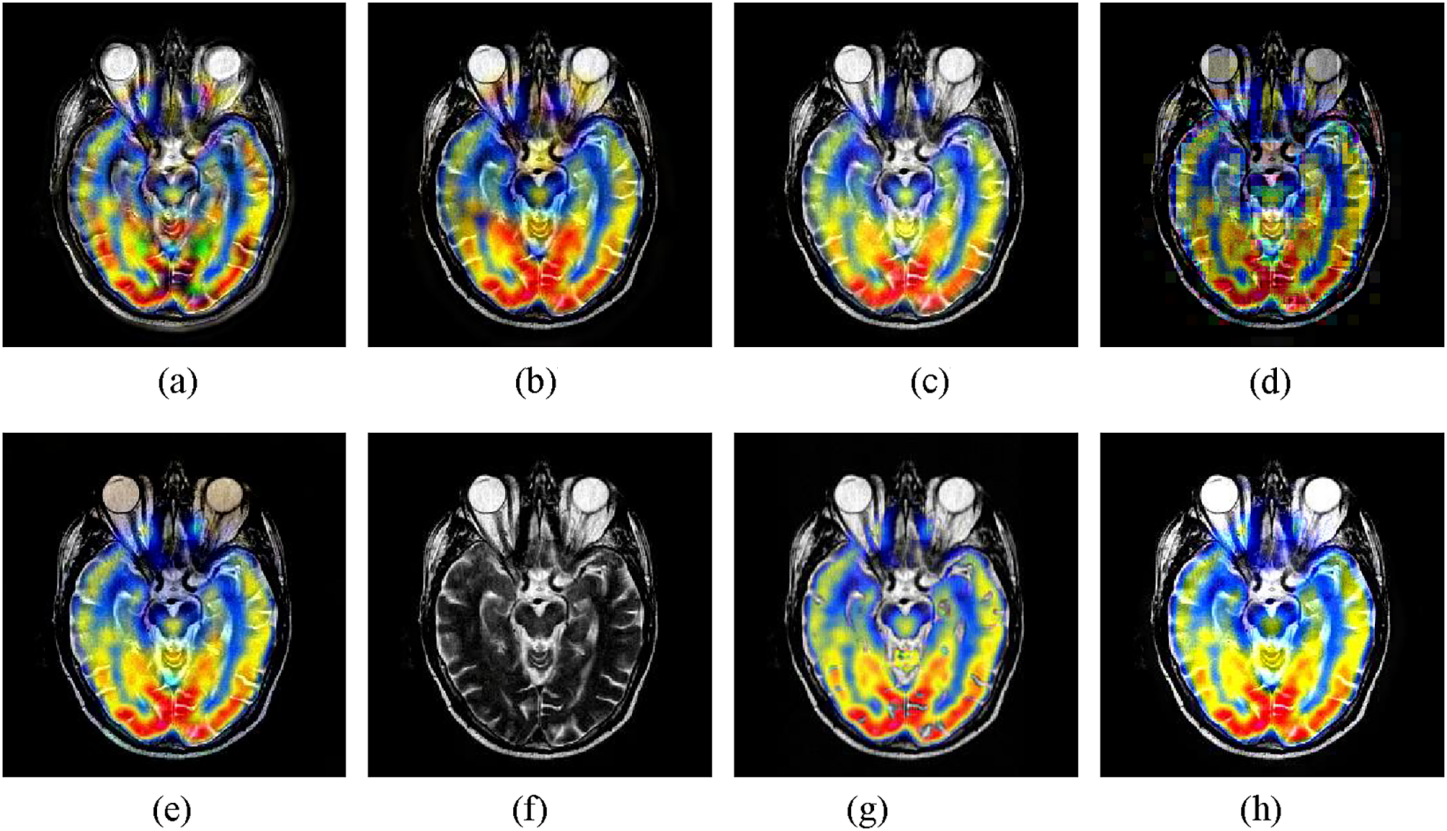
Fusion results of different methods for MRI and PET images. (**a**) CNN-NSCT. (**b**) NSST. (**c**) NSST-ST. (**d**) SR. (**e**) MST-SR. (**f**) GFF. (**g**) DSWT-RBFN. (**h**) Our method.

The second experiment is performed on the MRI and PET images. The fusion images yielded by different methods are shown in Fig. 4. In this example, the CNN-NSCT and NSST methods produce unsatisfactory performance caused by loss of energy. The NSST-ST method fails to preserve well the color information of the source PET image. The SR and GFF methods lead to severe color distortion. The resulting image of the DSWT-RBFN method cannot well retain the color information in the original PET image. In contrast, the proposed method can obtain higher visual effect in terms of both color preservation and detail extraction against other studied methods.

The average objective indexes of different approaches on 11 pairs of MRI and PET images are presented in Table 2. We can see that the proposed method still produces the best performance against other compared methods, which also further verifies the advantage of the proposed method. For running time of all methods, it can be seen that the GFF method is efficient since it only requires image decomposition and fusion without involving any deep features. The computing time of the proposed method is moderate among all approaches.

**Table 2:** Average objective indexes of different methods on 11 pairs of MRI and PET images.

Methods	SD	EN	Q_MI_	Q_PC_	Time(s)
CNN-NSCT	80.64	5.42	0.67	0.41	22.18
NSST	71.42	4.81	0.64	0.39	9.87
NSST-ST	80.56	5.27	0.71	0.47	11.29
SR	57.61	4.74	0.62	0.38	16.33
MST-SR	72.65	4.62	0.64	0.37	18.19
GFF	75.09	4.68	0.72	0.41	**2.43**
DSWT-RBFN	81.52	5.37	0.75	0.49	3.21
Our method	**83.52**	**5.64**	**0.77**	**0.51**	5.73

Significant values are in bold.

#### MRI and SPET image fusion

**Figure 5:**
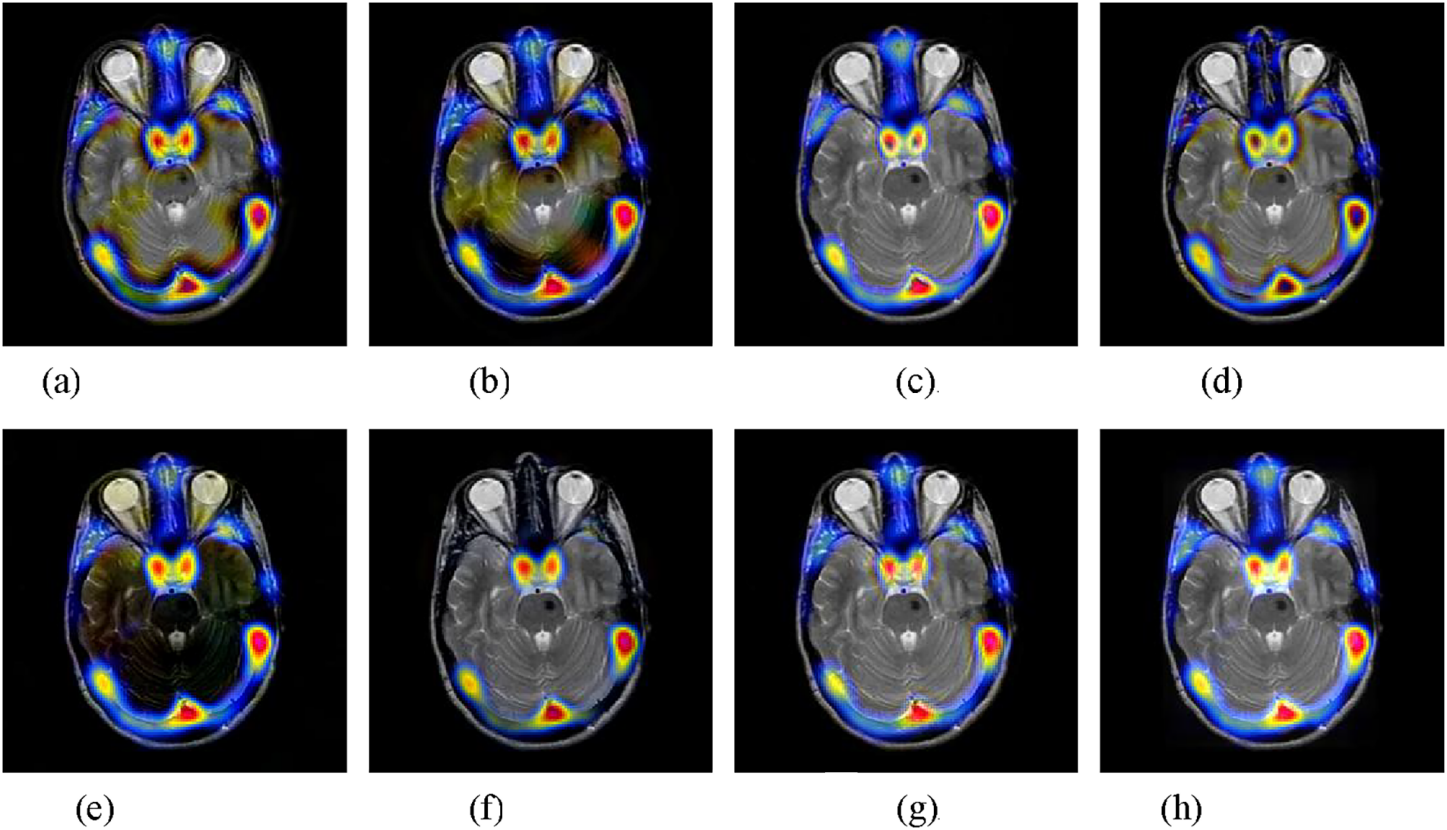
Fusion results of different methods for MRI and SPET images. (**a**) CNN-NSCT. (**b**) NSST. (**c**) NSST-ST. (**d**) SR. (**e**) MST-SR. (**f**) GFF. (**g**) DSWT-RBFN. (**h**) Our method.

The third experiment is tested on MRI and SPECT images. Figure 5 presents the fused images of different approaches. It is easy to observe that the proposed method still can retain the energy and details of source images. The compared methods cannot preserve color fidelity. Besides, the average objective indexes of different methods 11 pairs of MRI and SPET images are given in Table 3. It can be clearly observed that our method outperforms other studied methods on all the metrics, which is consistent with visual effect.

**Table 3:** Average objective indexes of different methods on 11 pairs of MRI and SPET images.

Methods	SD	EN	Q_MI_	Q_PC_	Time(s)
CNN-NSCT	63.41	4.87	0.72	0.45	22.09
NSST	58.24	4.67	0.56	0.43	9.77
NSST-ST	64.28	4.84	0.67	0.46	11.32
SR	59.71	4.52	0.71	0.42	16.78
MST-SR	55.35	4.31	0.57	0.35	18.43
GFF	60.08	4.67	0.62	0.37	**2.23**
DSWT-RBFN	65.41	4.98	0.78	0.45	3.38
Our method	**67.54**	**5.31**	**0.79**	**0.51**	5.81

Significant values are in bold.

#### MR-T1 and MR-T2 image fusion

**Figure 6:**
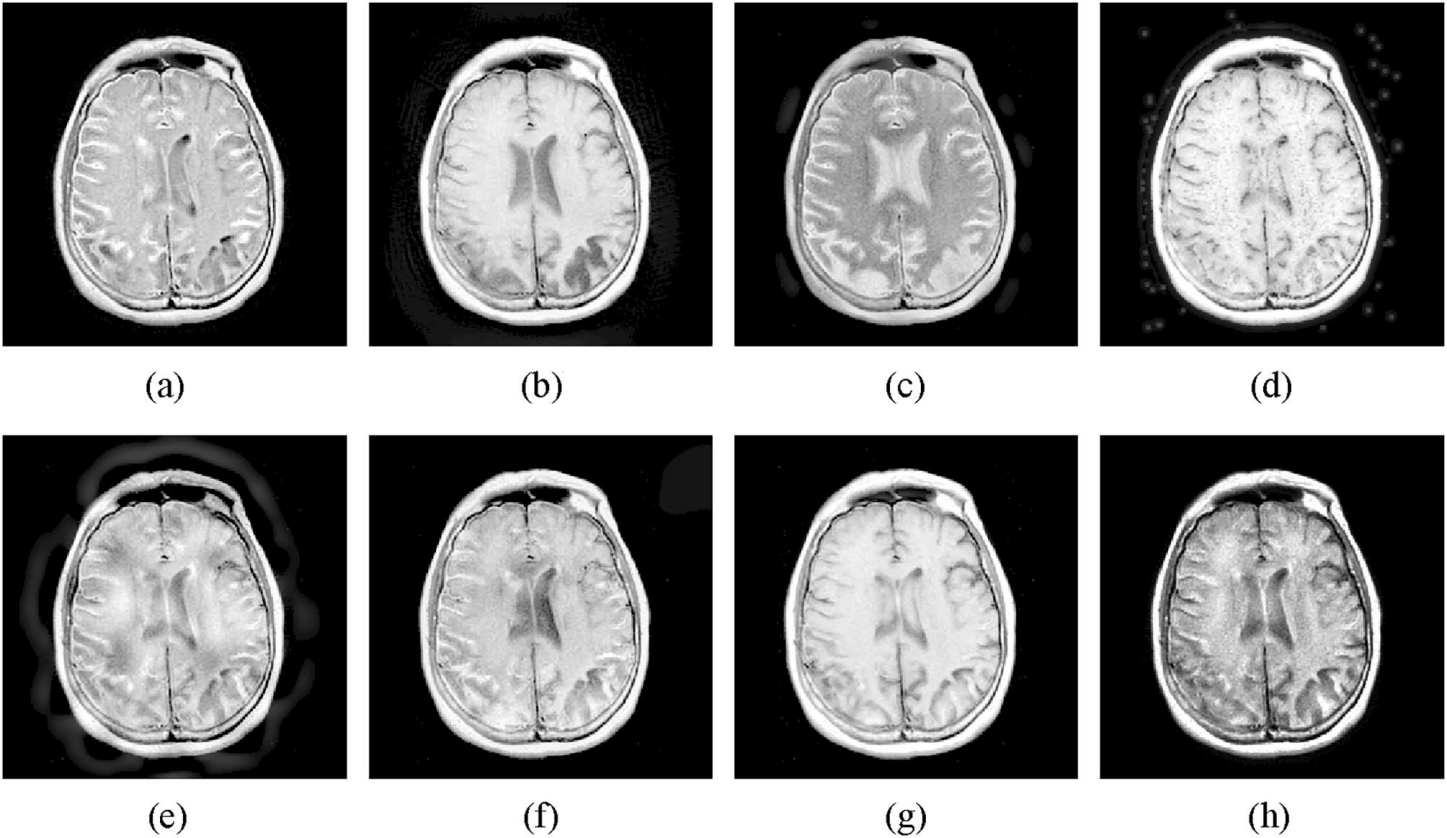
Fusion results of different methods for MR-T1 and MR-T2 images. (**a**) CNN-NSCT. (**b**) NSST. (**c**) NSST-ST. (**d**) SR. (**e**) MST-SR. (**f**) GFF. (**g**) DSWT-RBFN. (**h**) Our method.

The fourth experiment is conducted on MR-T1 and MR-T2 images. The fused results of different fusion approaches are presented in Fig. 6. The NSST-PCNN, SR, and MST-SR methods suffer from detail loss in the fused results. The NSST and DSWT-RBFN methods fail to well inject the MR-T2 image into the fused images. The GFF method cannot well integrate the details from MR-T1 image. By contrast, the proposed method can effectively merge the MR-T1 and MR-T2. Furthermore, Table 4 lists the objective metrics of all approaches on 11 pairs of MR-T1 and MR-T2 images. It is obvious that the proposed method still obtains the highest fusion results in terms of four indexes, which further illustrate the effectiveness of the proposed method.

**Table 4:** Average objective indexes of different methods on 11 pairs of MR-T1 and MR-T2 images.

Methods	SD	EN	Q_MI_	Q_PC_	Time(s)
CNN-NSCT	74.35	4.97	0.88	0.53	19.88
NSST	68.97	4.64	0.82	0.47	8.12
NSST-ST	69.87	4.62	0.84	0.49	9.13
SR	66.87	4.53	0.76	0.46	15.23
MST-SR	62.87	4.47	0.71	0.45	17.28
GFF	67.54	4.48	0.73	0.49	**1.42**
DSWT-RBFN	75.38	5.04	0.84	0.55	2.29
Our method	**80.37**	**5.16**	**0.86**	**0.57**	5.59

Significant values are in bold.

### Comparative study

In this subsection, several CNNs-based medical image fusion methods, including including transfer learning technique-guided VGG19 model (TL-VGG19)^43^, CNN-based fusion in NSST domain (NSST-CNN)^44^, asymmetric dual deep network with sharing mechanism (ADDNS)^45^, perceptual high frequency CNN (PHF-CNN)^37^, multiscale double-branch residual attention network (MSDRA)^46^, are adopted for comparison. An experiment is performed on 11 pairs of CT and MRI medical images. Table 5 presents the objective results of all compared methods. It can be observed that the proposed method still yields the highest fusion performance among all fusion techniques. This experiment also further verifies the effectiveness of the proposed method.

**Table 5:** Average objective results of different CNNs-based fusion methods on 11 pairs of CT and MRI images.

Methods	SD	EN	Q_MI_	Q_PC_
TL-VGG19	70.49	6.88	0.67	0.60
NSST-CNN	71.23	6.92	0.66	0.58
ADDNS	71.38	6.49	0.71	0.57
PHF-CNN	69.37	6.72	0.69	0.59
MSDRA	70.28	6.91	0.72	0.57
Our method	73.56	7.12	0.76	0.62

## Conclusions

In this work, a novel deep medical image fusion method based on a deep convolutional neural network (DCNN) is proposed for directly learning image features from original images. Specifically, source images are first decomposed by low rank representation to obtain the principal and salient components, respectively. Following that, the deep features are extracted from the decomposed principal components via DCNN and fused by a weighted-average rule. Then, considering the complementary between the salient components obtained by the low rank representation, a simple yet effective sum rule is designed to fuse the salient components. Finally, the fused result is obtained by reconstructing the principal and salient components. Experimental results verify that the proposed fusion method outperforms several state-of-the-art approaches. In future research work, the proposed method will be extended on multi-sensor image fusion.

## Data Availability

All images used in this work are collected from the database of Whole Brain Atlas created by Harvard Medical School (Available: http://www.med.harvard.edu/AANLIB/).
